# Place de la laparoscopie dans le diagnostic de la tuberculose péritonéale dans une région endémique de la Tunisie

**DOI:** 10.11604/pamj.2021.40.103.28568

**Published:** 2021-10-14

**Authors:** Issam Loukil, Yassin Maalej, Amine Zouari, Haythem Rjab

**Affiliations:** 1 Service de Chirurgie Générale Tataouine, Tataouine, Tunisie,; 2 Service de Chirurgie Générale Sfax, Sfax, Tunisie

**Keywords:** Tuberculose péritonéale, laparoscopie, biopsie, Peritoneal tuberculosis, laparoscopy, biopsy

## Abstract

La tuberculose reste un problème de santé publique en Tunisie. Le gouvernorat de Tataouine est une zone endémique. Le péritoine est atteint dans 1 à 2% des cas. Nous rapportons une étude épidémiologique rétrospective descriptive de 32 cas de patients opérés au Service de Chirurgie de Tataouine entre 2010 et 2020, la plupart par voie laparoscopique (28 patients), pour biopsie chirurgicale et confirmation histologique de tuberculose péritonéale. Nos patients étaient répartis en 24 femmes et 8 hommes soit un sexe ratio H/F de 0,33. L´âge médian était de 43 ans avec des extrêmes de 14 à 78 ans .La laparoscopie à visée diagnostique a été pratiquée dans 28 cas (87,5%). Devant l'aspect macroscopique per opératoire, le diagnostic de tuberculose péritonéale était fortement suspecté chez 16 patients (50%). L´étude anatomopathologique des biopsies réalisées a permis de confirmer le diagnostic chez tous les patients. Le but de ce travail est de rappeler les aspects épidémiologiques, cliniques de la tuberculose péritonéale dans une région endémique de la Tunisie et de présenter la place actuelle de la laparoscopie comme moyen diagnostic rapide et efficace.

## Abstract (en)

Tuberculosis is a public health problem in Tunisia. Tataouine governorate is an endemic area. Peritoneal involvement occurs in 1 - 2% of cases. We here report a retrospective, descriptive epidemiological study of 32 patients operated in the Department of Surgery at the Tataouine Hospital between 2010 and 2020. Laparoscopy was mainly performed (28 patients) to take biopsy samples for histologic confirmation of peritoneal tuberculosis. Twenty-four women and 8 men were involved in the study, with a M/F ratio of 0.33. The median age was 43 years, ranging from 14 to 78 years. Diagnostic laparoscopy was performed in 28 cases (87.5%). Given the intraoperative macroscopic aspect, the diagnosis of peritoneal tuberculosis was strongly suspected in 16 patients (50%). The anatomopathological study confirmed the diagnosis in all patients. The purpose of this study is to highlight the epidemiological, clinical features of peritoneal tuberculosis in an endemic region of Tunisia as well as the current role of laparoscopy as a rapid and effective diagnostic procedure.

## Introduction

La tuberculose reste de nos jours un problème de santé publique dans les pays en voie de développement et notamment en Tunisie. Son incidence a augmenté au cours des dernières années. Actuellement, un tiers de la population mondiale est infecté par le bacille tuberculeux avec 8 à 10 millions nouveaux cas/an et 3 millions de décès dans le monde [1]. La tuberculose péritonéale est une présentation rare de la tuberculose, elle présente 0,1 à 4% de toutes les localisations de la maladie tuberculeuse et pose encore des problèmes diagnostiques. La région de Tataouine au sud de la Tunisie est considérée comme endémique [2,3].

Le diagnostic reste difficile en raison du manque de la spécificité du tableau clinique, des examens biologiques et radiologiques et d´une faible sensibilité de l´isolement du *Mycobacterium tuberculosis* dans le liquide d´ascite d´autant plus les nouvelles techniques diagnostiques (Polymerase Chain Reaction (PCR)), dosage de l´Adénosine Désaminase (ADA) ne sont pas toujours utilisées, et ne sont pas disponibles [2]. La cœlioscopie exploratrice avec biopsie est une technique efficace pour la confirmation histologique. Sa spécificité et sa sensibilité sont très élevées avec respectivement 93% et 98% à travers la littérature [4]. Le but de cette étude est de décrire le profil épidémiologique, clinique et para clinique de la tuberculose péritonéale dans la région de Tataouine, dégager la sensibilité des examens clinique et para clinique, dégager la sensibilité de l´aspect macroscopique per opératoire et des résultats histologiques de la biopsie chirurgicale dans l´établissement du diagnostic positif.

## Méthodes

**Type de l´étude**: il s´agit une étude rétrospective descriptive.

**Cadre de l´étude**: elle a été menée au Service de Chirurgie Générale de la région de Tataouine en Tunisie durant la période de janvier 2010 à Décembre 2020.

**Participants à l´étude**: nous avons inclus tous les patients admis dans notre service au cours de la période d´étude ayant un diagnostic de tuberculose péritonéale retenu sur les résultats histologiques des biopsies chirurgicales. On a exclu tous les dossiers présentant des données manquantes.

**Collecte des données**: nous avons analysé les dossiers cliniques à l´aide d´une fiche d´exploitation regroupant les paramètres suivants : données épidémiologiques, cliniques, biologiques, radiologiques, opératoires et histologiques. La récolte des données a été faite sur une fiche d´enquête en respectant l´anonymat.

**Analyse des données**: les données sont analysées avec le logiciel SPSS 18.0. Les variables quantitatives qui suivent une distribution normale (gaussienne) ont été décrites en moyenne et celles de distribution non normale en valeur médiane. Les variables qualitatives ont été décrites en effectif et en pourcentage.

## Résultats

**Caractéristiques de la population d´étude**: sur une période de 11 ans, l´étude a colligé 32 patients répartis en 24 femmes et 8 hommes soit un sexe ratio H/F de 0,33. L´âge médian de nos malades était de 43 ans avec des extrêmes de 14 à 78 ans. Une seule patiente a eu un antécédent de tuberculose ganglionnaire et une seule patiente a eu un antécédent familial de tuberculose pulmonaire. Une forte consommation du lait cru et ses dérivés était noté chez 23 patients (71,9 %). Aucun de nos patients ne présentait une immunodépression.

**Eléments cliniques et para cliniques**: le délai de la consultation variait entre 1 jour et 5 mois avec une moyenne de 44 jours. Les motifs de consultation étaient dominés par les douleurs et l´ascite abdominale (Tableau 1). Seulement, huit patients ont eu une ponction exploratrice du liquide d´ascite. Le liquide d'ascite était du type exsudatif chez ces patients (Tableau 2). L´intra dermo-réaction à la tuberculine était positive chez 7 malades (21,9%). Les tests de PCR, de dosage de l´interféron gamma et de l´activité de l´adénosine désaminase dans le liquide d´ascite n´a pas été faite. Le dosage de CA 125 est augmenté pour 3 patients. La sérologie HIV a été réalisée chez 6 patients (18,7%) revenue négative. La tomodensitométrie abdominale a été pratiquée chez 28 patients (87,5%). Au terme de ce bilan clinique, biologique et radiologique, le diagnostic de tuberculose péritonéale était fortement suspecté pour 10 patients (31,25%).

**Tableau 1:** caractéristiques générales

Variables	Nombre	Pourcentage (%)
**Sexe**		
Homme	8	25
Femme	24	75
**Age**		
<40 ans	17	53,12
>40 ans	15	46,87
**Antécédents personnel de tuberculose**	1	3,12
**Consommation de lait cru**	23	71,87
**Motifs de consultation**		
Douleur abdominale	14	43,7
Ascite abdominale	13	40,6
Fièvre	7	21,8
**Signes physiques**		
Ascite abdominale	8	25
Douleur à la palpation	8	25
Pâleur cutanéo muqueuse	5	15,62

**Tableau 2:** caractéristiques cytochimiques du liquide d´ascite

Variables	Nombre des cas	Pourcentage(%)
**Aspect**		
Jaune citrin	6	75
Séro-hématique	1	12,5
Trouble	1	12,5
Purulent	0	0
Total	8	100
**Nombre de cellules/ml**		
<400 El/ml	2	25
>400 El/ml	6	75
Total	8	100
% de lymphocyte >60%	7	87,5
% de lymphocyte <60%	1	12,5
Total	8	100
**Taux de protide**		
< 25g/l	0	0
>25g/l	8	100
Total	8	100
RBK	0	0
Cellules atypiques	0	0

**Figure 1:**
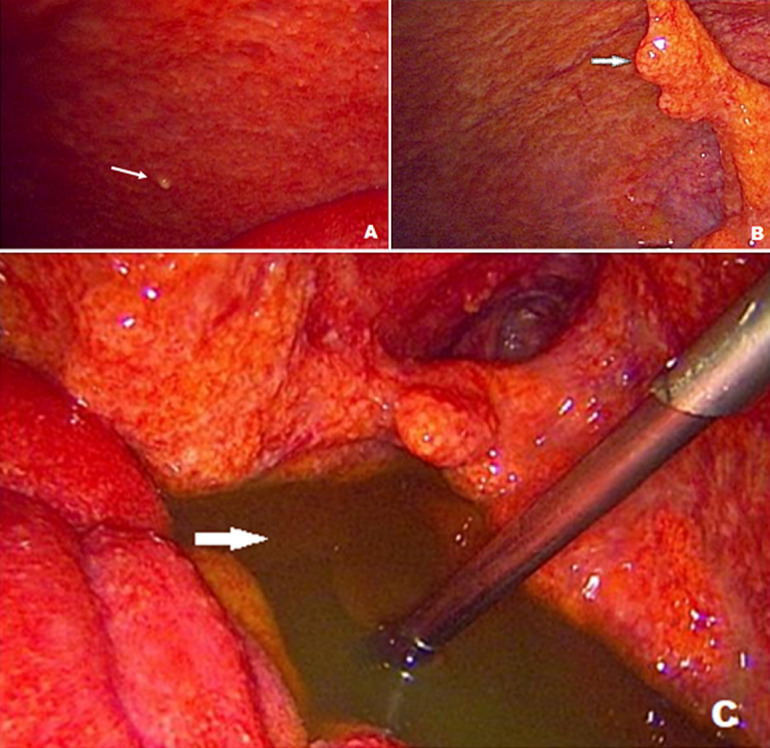
A) nodules péritonéaux, B) adhérences épiploon pariétales, C) prélèvement du liquide d´ascite

**Résultats opératoires**: la biopsie laparoscopique a été pratiquée dans 28 cas (87,5%) contre 4 cas de biopsie chirurgicale à ciel ouvert chez des patients présentant des antécédents de chirurgie abdominale. L´aspect macroscopique a été dominé par l´ascite abdominale et les nodules péritonéaux (Figure 1) (Tableau 3). Ainsi devant l'aspect macroscopique per opératoire, le diagnostic de tuberculose péritonéale était fortement suspecté chez 16 patients (50%).

**Tableau 3:** signes scannographiques et aspects macroscopiques per opératoires

Variables	Nombre	Pourcentage(%)
**Signes TDM**		
Ascite	20	62,5
Infiltration épiploïque nodulaire	13	40,6
Adénopathie à centre nécrosée	10	31,25
Nodules péritonéaux	7	21,8
**Aspects macroscopiques**		
Ascite	17	53,1
Nodules péritonéaux	16	50
Adhérences intra péritonéales	5	15,6
Inflammation péritonéale	4	12,5
Anses agglutinées	3	9,3

**Figure 2:**
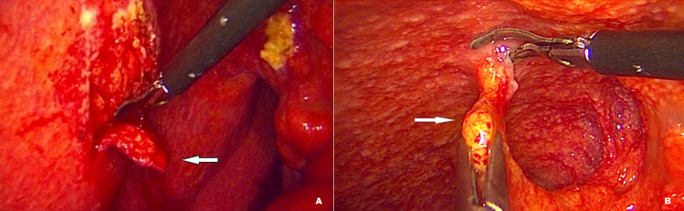
A) biopsie péritonéale, B) biopsie épiploïque

**Diagnostic positif et traitement**: le prélèvement du liquide d´ascite pour étude cytochimique et/ou bactériologique était réalisé chez 13 patients (40,6%), l´isolement du bacille de Koch dans le liquide est négatif. Des biopsies péritonéales et épiploïques étaient réalisés chez tous les patients (Figure 2). La présence de cellules géantes et de granulome épithelioïde avec ou sans nécrose caséeuse a permis de confirmer la tuberculose péritonéale chez tous les patients (Tableau 4).

**Tableau 4:** sensibilités diagnostiques des examens réalisés

Test réalisé	Sensibilité
Suspicion clinique, biologique et radiologique	31,25 %
Aspect macroscopique per opératoire	50 %
Etude histologique	100 %

## Discussion

Notre série montre un profil épidémiologique caractérisé par une prédominance féminine avec un sexe ratio H/F de 0,3 et un âge médian de 43 ans avec des extrêmes de 14 à 78 ans. L´analyse des résultats cliniques et para cliniques de nos patients a permis d´évoquer fortement le diagnostic positif de tuberculose péritonéale dans 31% des cas. La laparoscopie diagnostique a évoqué le diagnostic dans 50% des cas sur l´aspect per opératoire et de pratiquer des biopsies systématiques permettant la confirmation histologique.

Le nombre réduit de patients colligés représente la principale limite de notre étude. L´analyse du liquide de ponction d´ascite et l´intra dermo réaction à la tuberculine n´ont pas été généralisés à tous nos patients ce qui limite les éléments de présomption diagnostique. Aucun malade n´a bénéficié d´un dosage de l´adénosine désaminase dans le liquide d´ascite ni d´interféron gamma ni de test de réaction de polymérisation en chaîne. Ces examens sont de coût très élevés et ne sont pas disponible dans notre région.

Multiples moyens diagnostiques de la tuberculose péritonéale ont été décrits à travers la littérature. L´isolement du bacille de Koch dans le liquide d´ascite étant un bon moyen diagnostique, nécessite néanmoins une période d´attente de culture de 3 à 4 semaines [5]. Le dosage de l´adénosine désaminase dans le liquide d´ascite représente un test rapide et précis avec une sensibilité de 96% et une spécificité de 98% [6-8]. La réaction de polymérisation en chaîne est un test rapide permettant d´isoler le bacille de koch dans 24 à 48 heures avec une sensibilité de 60 à 80% et une spécificité de 96% [3]. Le dosage de l´Interféron Gamma (QuantiFERON) a une sensibilité de 93% et une spécificité de 99% notamment pour le diagnostic des formes latentes [9]. Le CA125 est un marqueur non spécifique de l´inflammation péritonéale, qui peut être augmenté (supérieur à35UI/ml) et peut atteindre des chiffres de 1400 UI/ml [10].

La tomodensitométrie peut montrer certains signes évocateurs : densité élevée de l´ascite, les adénopathies intra péritonéales avec centre hypo dense hypodense correspondant à la nécrose caséeuse, épaississement péritonéal avec réhaussement hétérogène, l´agglutination des anses et l´épaississement épiploïque qui peut être le plus souvent nodulaire, pseudo-tumoral ou ayant parfois un aspect de « gâteau épiploïque » [11]. Ces mêmes signes ont été analysés dans 87,5% des cas de notre série améliorant notre présomption diagnostique. La cœlioscopie exploratrice avec biopsie est une technique efficace surtout devant la non disponibilité des examens de diagnostic spécifiques déjà cités, tel est le cas dans notre région. C'est une technique qui permet l'exploration directe de la cavité péritonéale et la pratique systématique de plusieurs biopsies. Sa spécificité et sa sensibilité représentent respectivement 93% et 98% [12]. Les aspects macroscopiques sont principalement représentés par les nodules péritonéaux dans (76 à 100% des cas), très évocatrices de tuberculose lorsqu´elles sont millimétriques faisant 0,5 à 2 mm de diamètre, blanchâtres, de tailles généralement égales parsemant le péritoine pariétal et viscéral, et par les adhérences péritonéales et l´inflammation du feuillet péritonéal en second lieu [13,14]. Dans notre série ces constatations ont été marquées par la prédominance de l´ascite abdominale et des nodules péritonéaux dans plus de 50% des cas. L´apport de la laparoscopie dans le diagnostic de la tuberculose péritonéale pourrait être mieux évalué par des études de validation diagnostique de bonnes qualités et de fortes puissances.

## Conclusion

Les femmes jeunes représentent la majorité des patients touchés par la tuberculose péritonéale dans notre série. Les éléments cliniques et para cliniques aident à améliorer l´orientation étiologique. La laparoscopie diagnostique prend sa place devant la non disponibilité des tests biologiques spécifiques en augmentant la présomption diagnostique per opératoire et par la pratique des biopsies systématiques pour confirmation histologique.

### Etat des connaissances sur le sujet

- La tuberculose reste de nos jours un problème de santé publique dans les pays en voie de développement et notamment en Tunisie;
- La tuberculose péritonéale présente 0,1 à 4% de toutes les localisations de la maladie tuberculeuse;
- Le diagnostic reste difficile en raison du manque de la spécificité du tableau clinique, des examens biologiques et radiologiques.

### Contribution de notre étude à la connaissance

- Les éléments cliniques et para cliniques aident à améliorer l´orientation étiologique;
- La laparoscopie diagnostique prend sa place devant la non disponibilité des tests biologiques spécifiques en augmentant la présomption diagnostique per opératoire et par la pratique des biopsies systématiques pour confirmation histologique.

## Remerciements

On remercie Dr Ghassen Larbi et Dr Sana Benzarti pour leur aide précieuse.
